# Improvement in Violacein Production by Utilizing Formic Acid to Induce Quorum Sensing in *Chromobacterium violaceum*

**DOI:** 10.3390/antiox11050849

**Published:** 2022-04-26

**Authors:** Kuan-Chen Cheng, Hsiang-Chun Hsiao, Yu-Chen Hou, Chang-Wei Hsieh, Hsien-Yi Hsu, Hung-Yueh Chen, Shin-Ping Lin

**Affiliations:** 1 Graduate Institute of Food Science and Technology, National Taiwan University, 1 Roosevelt Rd., Sec. 4, Taipei 10617, Taiwan; kccheng@ntu.edu.tw (K.-C.C.); r08641005@ntu.edu.tw (H.-C.H.); hyc922@ntu.edu.tw (H.-Y.C.); 2 Institute of Biotechnology, National Taiwan University, 1 Roosevelt Rd., Sec. 4, Taipei 10617, Taiwan; 3 Department of Medical Research, China Medical University Hospital, China Medical University, 91 Hsueh-Shih Rd., Taichung 40402, Taiwan; 4 Department of Optometry, Asia University, 500, Lioufeng Rd., Wufeng, Taichung 41354, Taiwan; 5 Master Program in Food Safety, Taipei Medical University, 250 Wu-Hsing Street, Taipei 11042, Taiwan; ychou@tmu.edu.tw; 6 Department of Food Science and Biotechnology, National Chung Hsing University, 145 Xingda Rd., Taichung 40227, Taiwan; welson@nchu.edu.tw; 7 School of Energy and Environment, City University of Hong Kong, Kowloon Tong, Hong Kong 518057, China; sam.hyhsu@cityu.edu.hk; 8 Shenzhen Research Institute, City University of Hong Kong, Hong Kong 518057, China; 9 Department of Materials Science and Engineering, City University of Hong Kong, Kowloon Tong, Hong Kong 518057, China; 10 School of Food Safety, Taipei Medical University, 250 Wu-Hsing Street, Taipei 11042, Taiwan

**Keywords:** *Chromobacterium violaceum*, violacein, quorum sensing, formic acid, microbial pigment

## Abstract

Violacein has attracted increasing attention due to its various biological activities, such as antibacterial, antifungal, antioxidative, and antitumor effects. To improve violacein production, formic acid (FA) was added to a culture medium, which resulted in a 20% increase (1.02 g/L) compared to the no-FA-addition group (0.85 g/L). The use of a stirred-tank bioreactor system also improved violacein production (by 0.56 g/L). A quorum-sensing (QS)-related gene (*cviI*) was induced by FA treatment, which revealed that the mechanism induced by FA utilized regulation of the *cviI* gene to induce the *vio* gene cluster for violacein production. To analyze the antioxidative properties of the violacein produced, 2,2-diphenyl-1-picryl-hydrazyl-hydrate (DPPH) and 2,2′-azinobis-(3-ethylbenzthiazoline-6-sulphonic acid) (ABTS) scavenging tests were conducted, and results reveal that the values of the 50% inhibitory concentration (IC_50_) of DPPH and ABTS were 0.286 and 0.182 g/L, respectively. Violacein also showed strong inhibitory activity against Gram-positive bacteria (*Staphylococcus aureus* and *Bacillus subtilis*). In summary, this study found that the addition of formic acid can promote QS of *Chromobacterium violaceum*, thereby promoting the synthesis of violacein. Subsequently, the promoting effect was also evaluated in a bioreactor system. These findings will be helpful in establishing an economically beneficial production model for violacein in future work.

## 1. Introduction

Violacein is a purple pigment produced by microorganisms, such as species of the genera *Chromobacter*, *Pseudoalteromonas*, *Janthinobacterium*, and *Duganella* [1], which are often found in plants, soils, rivers, and marine environments [2]. *Chromobacterium violaceum (C. violaceum)*, a Gram-negative and facultative aerobic bacterium [3], has been extensively studied due to its high violacein production capacity [4]. Violacein presents various biological functions, such antibacterial [5], antifungal [6], antiviral [7], antiparasitic [8], and anticancer properties [9], which can be used in medical applications. On the other hand, microbially produced violacein is more in line with modern environmental protection concepts than chemically synthesized dyes are. Therefore, violacein has also garnered the attention of the textile industry [10].

In spite of violacein presenting high economic value, the low violacein yield of *C. violaceum* is considered to be the main limitation of its application [11]. Different strategies have been applied to increase its production. In the violacein biosynthesis pathway, tryptophan is an important substrate that can be added to media to significantly increase violacein production [12]. Some agricultural wastes, such as soybean meal [13], sugarcane bagasse [12], and pineapple waste [14], were also applied as alternative nutrients to decrease violacein production costs. In studies of violacein-producing strains, *Corynebacterium glutamicum* [15], *Yarrowia lipolytica* [16], and *Escherichia coli* [17] were designed as genetically modified strains using biosynthesis engineering technology. The violacein synthesis gene cluster, *vioABCDE,* was transformed into the hosts for violacein production. In addition, a bioreactor was used to scale up violacein production [17]. However, violacein is a water-insoluble compound, and accumulates inside bacteria [18], which may inhibit its production. This endo-secretion property makes it difficult to develop an immobilization system for continuous fermentation. Thus, improving the efficiency of present bioreactor systems has become an important issue for the industrial-scale production of violacein.

Quorum sensing (QS) is a well-known signaling mechanism in microorganisms for controlling various phenotypes, such as biofilm formation, swarming motility, H_2_O_2_ resistance, and cell aggregation [19]. In the violacein biosynthesis pathway of *C. violaceum*, QS also plays a crucial role. In the *C. violaceum* QS regulation system, homologous systems such as CviI/R and LuxI/R can be activated by utilizing the autoinducer, N-acyl-L-homoserine lactones (AHLs) [20], to induce violacein production. The released autoinducer accumulates in the environment outside cells, which depends on an increased cell density and further induces the QS system when the concentration of the autoinducer reaches a threshold. Therefore, regulation of a QS system using the addition of an autoinducer can be a means of improving violacein production [21]. However, autoinducers (e.g., AHLs) are expensive; hence, finding alternative QS inducers has become a potential direction for increasing violacein production.

Formic acid (FA) is a commonly used organic acid and has been applied in the agricultural, textile, and pharmaceutical industries for many years [22]. A previous study indicated that hydrolysis of agriculture wastes, such as sugarcane bagasse, can be used to obtain a carbon source from cellulose for fermentation applications. Nevertheless, some toxic compounds such as FA may also be released from the hydrolysis process [23]. During a pretest of applying agriculture waste hydrolysates to bacterial cellulose (BC) production [24,25], we noted that BC production improved with a specific concentration of FA. BC is considered to function as a kind of biofilm that can provide protection for bacteria against harm from heat, ultraviolet (UV) light, and antibiotics [26]. Consequently, this phenomenon provided a hypothesis that FA’s role could possibly be correlated with regulating a QS system. This study investigated the effect of adding FA to increase violacein production and examined the QS mechanisms involved. Furthermore, FA applied for violacein yield enhancement in a bioreactor system was also evaluated for developing an industrial violacein production process.

## 2. Materials and Methods

### 2.1. Materials

L-Tryptophan was purchased from Great Amino Trading (Kaohsiung, Taiwan). Beef extract was purchased from Biolife (Milan, Italy). Peptone was purchased from Bioshop (Burlington, ON, Canada). Tryptic soy broth (TSB) was purchased from Becton Dickinson (Franklin Lakes, NJ, USA). FA was purchased from Honeywell Fluka (Steinheim, Germany). Dimethyl sulfoxide (DMSO), 2,2′-azino-bis (3-ethylbenzothiazoline-6-sulphonic acid (ABTS), and 2,2-diphenyl-1-picrylhydrazyl (DPPH) were purchased from Merck (Darmstadt, Germany). Trolox was purchased from Sigma-Aldrich (St. Louis, MO, USA). Dulbecco’s modified Eagle’s medium (DMEM), fetal bovine serum (FBS), penicillin–streptomycin, and cell counting kit-8 (CCK-8) were purchased from GE Healthcare Life Science (Logan, UT, USA).

### 2.2. Microorganisms and Maintenance

*C. violaceum* ATCC 12472, *E. coli* BCRC 10239, Salmonella enterica serovar typhimurium (*S. typhimurium*) BCRC 10747, Staphylococcus aureus (S. aureus) ATCC 6538P, and Bacillus subtilis (*B. subtilis*) BCRC 17435 were purchased from the Bioresource Collection and Research Center (BCRC; Hsinchu City, Taiwan). These bacteria were kept frozen in double-distilled (dd)H_2_O containing 20% (*v/v*) glycerol at −80 °C. *C. violaceum* was grown routinely in nutrient broth (NB) containing 5 g/L peptone and 3 g/L beef extract at 25 °C with shaking at 180 rpm in the dark.

*C. violaceum* was inoculated on nutrient agar and incubated at 25 °C. Colonies of *C. violaceum* were inoculated into 250 mL flasks containing 10 mL of sterilized NB and incubated at 25 °C and 180 rpm until the absorbance reached an approximate OD_600_ value of 1.1. Subsequently, a final volume of 1% inoculum of bacterial culture was transferred to 250 mL flasks containing 10 mL of sterilized NB with different concentrations of FA (40, 80, and 160 ppm) and L-tryptophan (0.075, 0.15, 0.3, 0.6, and 1.2 mg/mL) and incubated at 25 °C and 180 rpm for 48 h. After incubation, the cell number and the amount of crude violacein were determined.

The biomass of *C. violaceum* was determined according to a previously reported method [27] with slight modifications. Briefly, 1 mL of bacterial culture was transferred to a 1.5 mL Eppendorf tube and centrifuged at 10^4^× *g* for 10 min. The supernatant was discarded, and 1 mL of DMSO was added to the pellet. After vortexing for 10 min with a vortex mixer, the DMSO was removed, and the pellet was resuspended in 1 mL of ddH_2_O to measure the absorbance at 600 nm using a UV/visible (vis) spectrometer (Mecasys, Daejeon, Korea). The biomass of *C. violaceum* was calculated via linear regression. The correlation between the OD_600_ value and cell number was determined by a plating method to calculate the colony-forming units (CFU) of *C. violaceum*.

### 2.3. Violacein Production and Its Measurement

Violacein was extracted according to a previously reported method [28] with slight modifications. Briefly, 1 mL of the bacterial culture was transferred to a 1.5 mL Eppendorf tube and centrifuged at 10^4^× *g* for 10 min. The resulting supernatant was discarded, and 1 mL of DMSO was added to the pellet. After vortexing, cells were disrupted by ultrasonication for 15 min. The DMSO extract was then separated from cells by centrifugation at 10^4^× *g* for 7 min. The amount of crude violacein in the supernatant was determined by measuring the absorbance at 575 nm with a UV/vis spectrometer (Mecasys). The concentration of violacein was calculated using the Beer–Lambert Law, and the molar extinction coefficient of violacein used in this experiment was 10.955 L/g/cm [29]. The concentration of violacein was calculated using the following Equation:

A=εbc;

where A is the absorbance of the sample at 575 nm, ε is the molar extinction coefficient of violacein, b is the length of the light path, and c is the concentration of the sample.

### 2.4. Production of Violacein in the Stirred-Tank Bioreactor

Violacein was produced in a 3.5 L stirred-tank bioreactor (Firstek, New Taipei City, Taiwan) in batch mode. A final volume of 1% inoculum of *C. violaceum* was transferred to 250 mL flasks containing 50 mL of sterilized NB and incubated at 25 °C and 180 rpm for 18 h in the dark. Subsequently, 200 mL of bacterial culture was transferred to the bioreactor containing 1800 mL of sterilized NB with 0.3 mg/mL L-tryptophan and 160 µg/mL FA and incubated for 48 h under the following conditions: 25 ± 3 °C, 180 rpm, an aeration rate of 1 vvm, and pH 7 ± 0.3. NB with only 0.3 mg/mL of L-tryptophan was used as a control. The pH value was maintained at 7 ± 0.3 with 1 N HCl. During fermentation, the glass tank of bioreactor was covered with aluminum foil sheets to prevent light from influencing violacein production.

### 2.5. Relative Expression of QS Genes Using a Quantitative Polymerase Chain Reaction (qPCR)

The QS system for *C. violaceum* was regulated by AHL and the *C. violaceum* QS receptor (CviR) receptor. To analyze QS in *C. violaceum*, a qPCR was used to investigate the gene expression of *cviI* coding for AHL synthase and *cviR* coding for the CviR.

Total RNA was extracted from *C. violaceum* using TRIzolTM (Invitrogen, Carlsbad, CA, USA) according to the manufacturer’s protocol, and then messenger (m)RNA was reverse-transcribed into complementary (c)DNA using RevertAid First Strand cDNA Synthesis (Thermo Scientific, Waltham, MA, USA). Then, 18 ng of cDNA was used to analyze gene expression using SYBR Green Real-Time PCR (Geneone, Seoul, Korea). qPCR conditions were as follows: 2 min at 50 °C, 10 min at 95 °C, and 40 cycles of 15 s at 95 °C and 60 s at 60 °C. The qPCR was performed using designed forward (F) and reverse (R) primers (*rpoB*-F, 5′-GCCCACACTTCCATCTCACCGAAAC-3′ and *rpoB*-R, 5′-TCCAAGACCCAGATGACCCTGTTCG-3′; *vioA*-F, 5′-CCTTGCCATGCTCTTTCAGC-3′ and *vioA*-R, 5′-CGAGGTGTATCCGTTCACCC -3′; *cviI*-F, 5′-GAAACCGTCCTCGCATAAGG-3′ and *cviI*-R, 5′-CTGAAACTAAGCTGCGACAGTTG-3′; and *cviR*-F, 5′-GGTATTGGGACGCCTGAACA-3′ and *cviR*-R, 5′-CTGGGAGTACTGGTTGAGCC-3′. The abundance was normalized to the *rpoB* housekeeping gene [30].

### 2.6. Antimicrobial Activity

To evaluate the antimicrobial activity, we investigated violacein’s actions against bacterial pathogens using a disc diffusion assay and minimum inhibitory concentration (MIC) assay. The bacterial pathogens we used in this experiment included *E. coli*, *S. aureus*, *B. subtilis*, and *S. typhimurium*. *E coli*, *S. aureus,* and *B. subtilis* were cultured in NB, while *S. typhimurium* was cultured in TSB.

A disc diffusion assay was used to examine the antibacterial effects of violacein as described by Sewify et al. [31] but with the following modifications. Briefly, 0.1 mL of overnight bacterial culture was inoculated on agar and spread over the surface of the agar using a sterile L-shaped spreader. Using a sterile cork-borer 5 mm in diameter, three holes per plate were made in the inoculated agar. Different concentrations of violacein at 40 μL, each diluted with DMSO were added to each well. The plates were then incubated at 37 °C for 24 h. After incubation, the diameters of the clear inhibition zones were measured to evaluate the antimicrobial activity. The same solvents utilized to dissolve violacein were used as negative controls.

### 2.7. Antioxidant Activity

#### 2.7.1. DPPH Radical Scavenging Activity

The scavenging capability of violacein against DPPH radicals was evaluated using a previously reported method [32] with slight modifications. Briefly, 20 μL of a sample was added to 180 μL of a 100 µM DPPH methanol solution in a 96-well plate. The absorbance at 517 nm was measured after 30 min at room temperature in the dark. In this assay, the same solvents utilized to dissolve violacein served as a control. The DPPH radical scavenging activity was calculated as follows:

$$\mathrm{DPPH}\;\mathrm{radical}\;\mathrm{scavenging}\;\mathrm{activity}\;\left(\%\right)=\left\{\frac{\left[\mathrm{Ac}-\left(\mathrm{As}-\mathrm{Ab}\right)\right]}{\mathrm{Ac}}\right\}*100;$$

where Ac is the absorbance of the DPPH methanol solution without a sample, As is the absorbance of the DPPH methanol solution and sample, and Ab is the absorbance of the sample without the DPPH methanol solution. The antioxidant activity of the sample was expressed as the concentration of a sample that caused 50% inhibition (IC_50_) of DPPH radicals, which was calculated via linear regression.

#### 2.7.2. ABTS Radical Scavenging Activity

The scavenging capability of violacein against ABTS^+^ radicals was evaluated using a previously reported method [32] with slight modifications. An ABTS^+^ stock solution was prepared by mixing equal volumes of 2.45 mM potassium persulfate in an aqueous solution and 7 mM ABTS in an aqueous solution and then incubating the mixture for 12 h in the dark to generate ABTS^+^ radicals. Then, the ABTS^+^ stock solution was diluted with ddH_2_O to an absorbance of 0.7 (±0.02) at 734 nm. Briefly, 20 μL of the sample was added to 180 μL of the ABTS^+^ solution in a 96-well plate. The absorbance at 734 nm was measured after 6 min at room temperature in the dark. In this assay, the same solvents utilized to dissolve violacein served as a control, and the sample and controls were examined in triplicate. The ABTS^+^ radical scavenging activity was calculated as follows:

$$\mathrm{ABTS}\;\mathrm{scavenging}\;\mathrm{activity}\;\left(\%\right)=\left\{\frac{\left[\mathrm{Ac}-\left(\mathrm{As}-\mathrm{Ab}\right)\right]}{\mathrm{Ac}}\right\}*100;$$

where Ac is the absorbance of the ABTS^+^ solution without a sample, As is the absorbance of the ABTS^+^ solution and sample, and Ab is the absorbance of a sample without the ABTS^+^ solution. The antioxidant activity of a sample was expressed as the concentration that caused 50% inhibition (IC_50_) of ABTS^+^ radicals, which was calculated via linear regression.

### 2.8. Statistical Analysis

Statistical evaluations of all experimental data (variation from basal values) were performed using an analysis of variance (ANOVA). Post hoc comparisons with the negative control were performed with Tukey’s test. Statistical analyses were conducted with IBM SPSS Statistics 19 (IBM, Armonk, NY, USA), and p<0.05 was considered significant.

## 3. Results and Discussion

### 3.1. Impacts of FA and Tryptophan Addition on Violacein Production by C. violaceum

**Figure 1:**
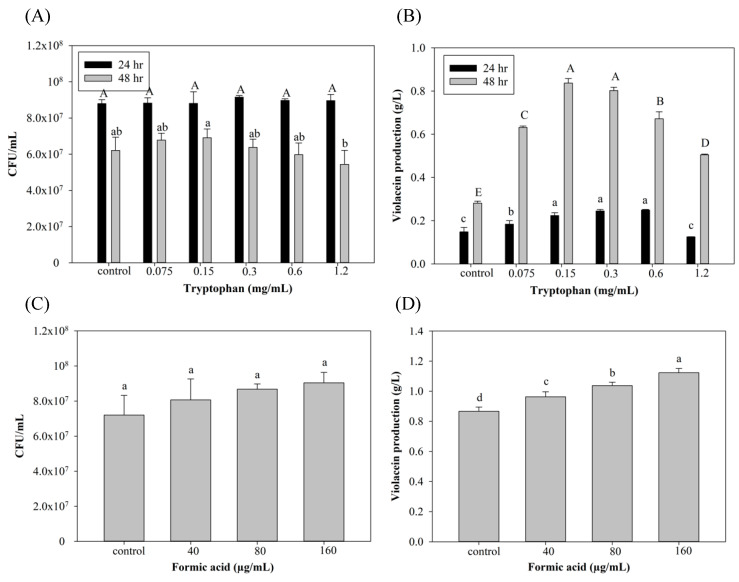
Effect of (**A**,**B**) tryptophan and (**C**,**D**) formic acid on cell growth and violacein production of *C. violaceum*. Each value is expressed as mean ± standard deviation (*n* = 3). Different superscripts are significantly different (*p* < 0.05).

Tryptophan is the substrate of violacein biosynthesis in *C. violaceum*. To optimize violacein production, concentrations of tryptophan and FA were evaluated, and results are shown in Figure 1, which demonstrates that the group with the addition of 0.15 and 0.3 mg/mL tryptophan presented the highest violacein production (0.83 and 0.8 g/L) with 196% and 185% improvements, respectively, after 48 h of culture compared to the control group. However, the addition of tryptophan at more than 0.3 g/L may have decreased violacein instead. This may have been due to unbalanced carbon/nitrogen ratios that disrupted tryptophan metabolism for violacein-producing microorganism growth [32]. The condition of 0.3 mg/mL tryptophan with 48 hr cultivation was used in the formic acid induction experiments (Figure 1C,D) due to its more stable violacein production. As to the addition of FA (Figure 1C,D), violacein production increased depending on the added FA concentration. However, cell death with no violacein production was seen after the addition of 320 µg/mL FA. Previous studies found that FA may exhibit toxicity toward microorganisms and can further inhibit the production of metabolic products [23,25]. There are no related studies demonstrating that FA may also play a role as an inducer. Interestingly, Liu et al. [33] found that the addition of 1/6 of the minimum inhibitory concentration (MIC) of kanamycin (the MIC of kanamycin in *C. violaceum* ATCC12472 was 8 µg/mL) induced QS of *C. violaceum*, resulting in an increase in violacein production. This finding suggests the possibility of an induction mechanism of FA in violacein production.

**Figure 2:**
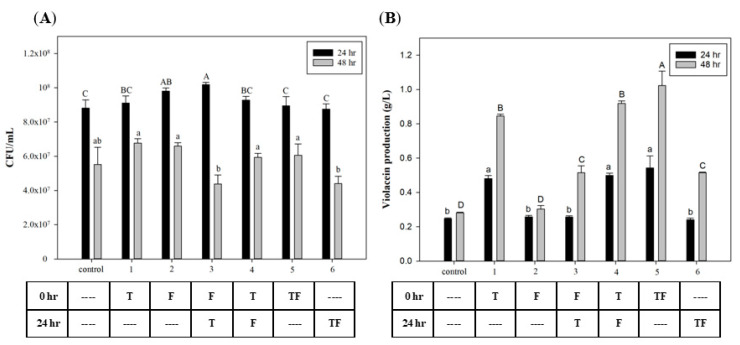
Effect of adding tryptophan and formic acid at different time points on (**A**) cell growth and (**B**) violacein production of *C. violaceum*. The different addition timing permutations of tryptophan and formic acid are presented in the table below the graph. T: 0.3 mg/mL tryptophan; F: 160 µg/mL formic acid. Each value is expressed as mean ± standard deviation (*n* = 3). Different superscripts are significantly different (*p* < 0.05).

To investigate the effect of the time that FA and tryptophan were added, different time points were used for biomass growth and violacein production. Figure 2A shows that the *C. violaceum* growth was highest in all groups after 24 h of incubation. In the growth phase of *C. violaceum,* cell growth rapidly increased after 12 h and entered the mid-log phase after 24 h [34]. Results show no effects on cell growth after the addition of tryptophan and FA at different time points. However, in the groups with initial tryptophan addition, violacein production had significantly increased at both 24 and 48 h later. These results suggest that using tryptophan as a substrate for violacein biosynthesis requires time for its conversion. The highest yield appeared in the group in which tryptophan and FA were added at 0 h, which reached 1.02 g/L. Figure 2B also reveals that the addition of FA improved violacein production (compared to groups 1 to 4 and 5) by 8.2% and 20%, respectively. This suggests that the effect of FA may begin after its addition, but adding tryptophan at the initial time point will provide greater induction than the sequential addition after 24 h.

### 3.2. Improvement in Violacein Production Using FA in a Bioreactor System

**Figure 3:**
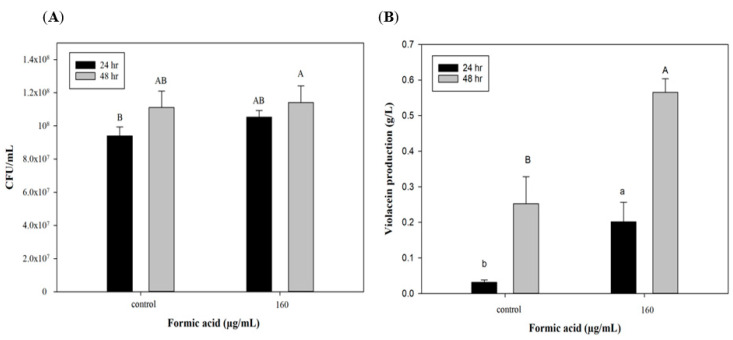
Effect of formic acid on (**A**) biomass and (**B**) violacein production of *C. violaceum* in bioreactor. Each value is expressed as mean ± standard deviation (*n* = 3). Different superscripts are significantly different (*p* < 0.05).

To further explore the potential of FA induction in scaling up production, a 3.5 L stirred-tank bioreactor was used with optimized culture conditions for violacein production. Figure 3A indicates that cell growth could reach 9 × 10^7^ CFU/mL after 24 h, which was the same as the flask system. However, cell numbers of *C. violaceum* had increased to 1.1 × 10^8^ CFU/mL in the bioreactor system by 48 h, which was in contrast to the results of the flask system. In the flask shaker system, a lack of nutrients and the accumulation of produced acids and metabolic products may have caused cell death after 48 h. However, in the reactor system, the culture medium was maintained at pH 7 and continuously provided with high dissolved oxygen concentrations leading to extended cell growth. This may explain why the trends of cell growth at 48 h of incubation differed in the two production systems. As to violacein production, the group with the FA addition exhibited significant increases of approximately 0.2 and 0.57 g/L after 24 and 48 h of culture, which were higher than values of the control group (0.03 and 0.25 g/L). These results show that the efficiency of violacein production in the bioreactor was lower than that of the flask system (lower by 50%, 0.56 vs. 1.12 g/L). Miao et al. [35] used a 10 L stirred-tank bioreactor to produce secondary metabolic products (triptolide, wilforgine, and wilforine) from *Tripterygium wilfordii*, and found that their production subsequently decreased to 6.48%, 6.27%, and 4.90% compared to the flask system. The authors mentioned that the initial inoculation and dissolved oxygen volume were both important for secondary metabolite production and should be optimized in the bioreactor system. In spite of lower violacein production, that bioreactor is still a necessary production tool for scaling up production due to its large volume. Furthermore, the addition of FA can produce a significant improvement in violacein production in the reactor system (Figure 3B). On the other hand, when improving the violacein yield by utilizing the addition of FA, there is no need to change the production system, which means that producers can save the cost of a system redesign.

### 3.3. Effects of FA on QS-Related Genes of C. violaceum

The violacein biosynthesis of *C. violaceum* is related to QS. During the production process, various gene clusters are involved in the violacein production pathway, including the *luxIR*, *cviIR,* and *vioABCDE* gene cluster [36]. Therefore, QS-related gene expressions were investigated to clarify the relationship of the FA induction mechanism with violacein production.

**Figure 4:**
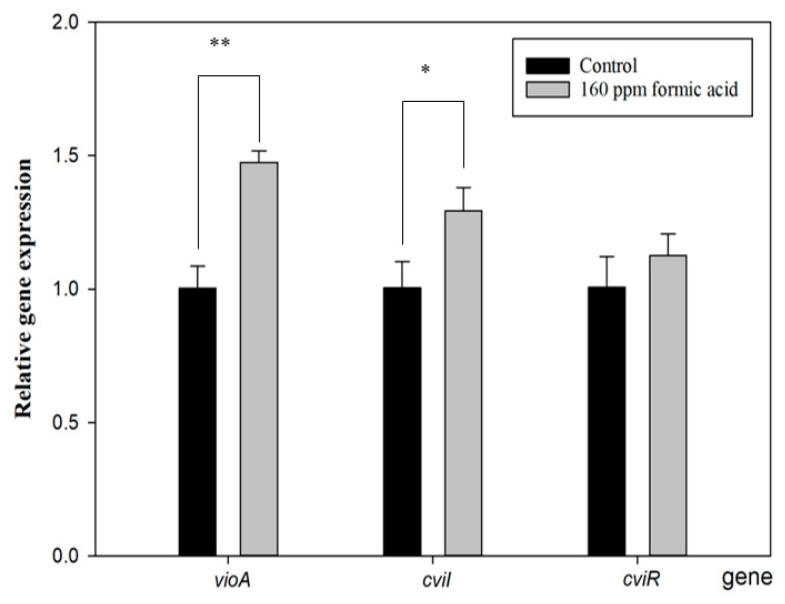
Effect of formic acid on the gene expression of *vioA*, *cviI*, and *cviR* in *C. violaceum*. Each value is expressed as mean ± standard deviation (*n* = 3). Asterisk represents a significant difference (* *p* < 0.05, ** *p* < 0.01).

Figure 4 shows that *cviI* gene expression increased with FA treatment. The function of CviI is related to the production of the autoinducer, C_10_-homoserine lactone (C_10_-HSL). When C10-HSL is released from bacteria and accumulates beyond the QS threshold, it will be detected by the violacein production receptor, resulting in violacein production [36]. Furthermore, the *vioA* gene expresses a rate-limiting step of the violacein synthesis pathway [37], and it was also enhanced with FA treatment. However, its upstream-regulated gene, *cviR,* did not show a difference compared to the control group. These results are similar to those of a previous study [33] and seem to indicate that FA directly regulates the *cviI* gene to induce the *vio* gene cluster for violacein production. Proving this assumption would require further direct evidence.

### 3.4. Antimicrobial Properties of the Produced Crude Violacein

The antimicrobial properties of crude violacein against *E. coli*, *S. aureus*, *B. subtilis,* and *S. typhimurium* were evaluated. In the inhibition zone test (Table 1), 0.13 mg/mL crude violacein was found to inhibit *S. aureus* and *B. subtilis* (with respective inhibition zones of 8.8 ± 0.4 and 9.6 ± 0.4 mm), but without antimicrobial activity against *E. coli* or *S. typhimurium.* Wang et al. [38] demonstrated that the ethanol extract of crude violacein exhibited strong antimicrobial properties against Gram-positive bacteria, such as *S. aureus* ACCC 10499, *B. subtilis* ACCC 10243, and *Bacillus megaterium* ACCC 01509, but exhibited no inhibition against Gram-negative bacteria (*E. coli* ACCC 10034, *Flavobacterium oryzae* ACCC 10051, or *Xanthomonas campestris* ACCC 10491). Because results of the inhibitory zone test may have been influenced by the culture temperature, the addition of samples, the thickness of the culture agar, and the nutrient composition of the agar [39], it was necessary to use another test to evaluate the antimicrobial ability of the produced violacein. The MIC test (Table 1) showed that crude violacein exhibited strong inhibitory actions against *S. aureus* and *B. subtilis* with MIC values of <0.01 and 0.03 g/L, respectively. Violacein exhibited weak inhibition against *S. typhimurium* and *E. coli* growth (with MIC values of 0.29 and >1 g/L). These MIC results are similar to results of the inhibition zone and a previous study [5]. Cox and Wright [40] indicated that the specificity of an antimicrobial reagent may be due to different membrane structures of Gram-negative and Gram-positive bacteria. Gram-negative bacteria have one more outer membrane that contains saturated fatty acids that decrease the membrane fluidity, which prevents antimicrobial reagents from entering the bacteria.

### 3.5. Antioxidant Properties of the Produced Crude Violacein

The antioxidant properties of crude violacein were estimated utilizing two kinds of radical scavenging assays (DPPH and ABTS assays). The DPPH results are shown in Table 2, and the IC_50_ of trolox (0.0936 g/L) was lower than that of crude violacein (IC_50_ of 0.2867 g/L), which suggests that the antioxidant ability of trolox was better than that of crude violacein. The ABTS assay also showed similar results (IC_50_ values of trolox and crude violacein were 0.0646 and 0.1822 g/L, respectively). Cao et al. [41] mentioned that specific N–H bonds of violacein present lower bond dissociation energy than N–H bonds of DPPH or ABTS. That is why violacein provides a scavenging ability to remove these radicals. Although the antioxidative ability of violacein was slightly lower than that of trolox, it still exhibited strong antioxidant properties. In particular, the violacein we used was a crude extract, which means that its antioxidant ability could be increased by purification [42].

## 4. Conclusions

This study evaluated the effect of formic acid on violacein production by *C. violaceum*. The violacein production increased by 20% with induction of 160 µg/mL formic acid and the addition of 0.3 mg/mL tryptophan. These optimal conditions were also applied to a stirred-tank bioreactor for a 50% increase in the violacein yield. Furthermore, the relationship of formic acid treatment and quorum sensing in violacein biosynthesis was clarified. Finally, the produced violacein exhibited great antioxidative and antimicrobial activities against Gram-positive bacteria. These findings suggest that the addition of formic acid can serve as a strategy for improving violacein production.

## Figures and Tables

**Table 1:** Antimicrobial properties of crude violacein on tested microbes.

Tested Microbes	MIC (g/L)		Inhibition Zone (mm)
	Ampicillin	Crude Violacein	Crude Violacein (0.13 g/L)
*S* *. aureus*	<0.01	<0.01	8.8 ± 0.4
*B. subtilis*	<0.01	0.03	9.6 ± 0.4
*S. typhimurium*	<0.01	0.29	--
*E* *. coli*	<0.01	>1	--

Values represent the mean of three replicates. MIC, minimum inhibitory concentration.

**Table 2:** Antioxidant effect of violacein against DPPH and ABTS radicals.

Sample	Conc. of Sample (g/L)	Inhibition (%)	Conc. of Sample (g/L)	Inhibition (%)
	DPPH assay		ABTS assay	
Trolox	0.08	42.37 ± 0.09	0.04	36.09 ± 0.4
	0.1	53.99 ± 0.42	0.05	41.23 ± 0.35
	0.12	62.84 ± 0.86	0.06	47.67 ± 0.47
	0.14	71.06 ± 0.2	0.07	53.14 ± 0.47
	0.16	77.04 ± 0.45	0.08	58.66 ± 0.94
	0.0936	IC_50_	0.0646	IC_50_
Crude violacein	0.1575	34.73 ± 0.6	0.105	34.30 ± 0.23
	0.21	42.59± 0.18	0.1313	39.45 ± 0.26
	0.2625	47.57 ± 0.18	0.1575	44.55 ± 0.72
	0.315	52.92± 0.2	0.1838	50.72 ± 0.91
	0.3675	58.71 ± 0.56	0.21	55.61 ± 0.75
	0.286	IC_50_	0.182	IC_50_

Values represent the mean of three replicates. IC_50_, 50% inhibitory concentration.

## Data Availability

Data are contained within the article.
